# The Presence of Consciousness in Absence Seizures

**DOI:** 10.3233/BEN-2011-0318

**Published:** 2011-03-29

**Authors:** Tim Bayne

**Affiliations:** Faculty of PhilosophyUniversity of Oxford & St. Catherine’s CollegeOxfordUK

**Keywords:** Consciousness, absence seizures, unity of consciousness, neural correlates of consciousness, background states of consciousness, contents of consciousness

## Abstract

This paper examines three respects in which the study of epileptic absence seizures promises to inform our understanding of consciousness. Firstly, it has the potential to bear on debates concerning the behavioural and cognitive functions associated with consciousness. Secondly, it has the potential to illuminate the relationship between background states (or ‘levels’) of consciousness and the contents of consciousness. Thirdly, it has the potential to bear on our understanding of the unity of consciousness.

## 1. Introduction

Although the study of epileptic absence seizures has the potential to contribute a great deal to the scientific understanding of consciousness, this potential has yet to be fully exploited. There have been a number of insightful discussions of consciousness in the context of epileptic seizures [12,17,22,23,33], but the basic conceptual issues are still poorly understood and many empirical questions remain unexplored. In this paper I review a number of questions that are of interest to consciousness scientists and identify ways in which the study of absence seizures might bear on their investigation.

The most fundamental question here is whether patients in the grip of an absence seizure are conscious in any manner at all. The following section examines this question, and provides a tentative case for thinking a number of such patients are conscious, if only in a minimal form. Subsequent sections examine ways in which the study of absence seizures might bear on three important questions that currently face the science of consciousness. Firstly, what is the relationship between the various cognitive and behavioural capacities associated with consciousness? Can these capacities dissociate from each other, or are the correlations between them unbreakable? Secondly, what is the relationship between background states (or 'levels') of consciousness and the contents of consciousness? How do the neural correlates underlying these two aspects of consciousness interact with each other? Thirdly, is consciousness necessarily unified? More specifically, might the unity of consciousness break down in certain kinds of absence seizure? This paper does not provide detailed answers to these questions, but instead charts the conceptual landscape in which attempts to answer these questions might be located.

## 2. Consciousness in absence seizures

Absence seizures are so-called because the patient appears to be 'absent' or disengaged from their immediate environment. Patients will typically fail to understand or respond to questions or commands and may have amnesia for the period in question. Absences can occur in the context of both partial (or focal) seizures, which arise in a circumscribed region of the brain (often the temporal lobe), and generalized seizures, in which

ISSN 0953-4180/11/$27.50 © 2011 – IOS Press and the authors. All rights reserved the seizure permeates widespread regions of the brain. Those that occur in the context of generalized seizures are often very brief (lasting for 5–10 seconds), whereas those that occur in the context of partial seizures – often called 'complex partial seizures' – can last for minutes.

Consciousness scientists generally assume that patients undergoing an absence seizure are no longer conscious [31,32]. However, there is ample reason to doubt whether such claims are correct. Consciousness is clearly severely *disrupted* in absence seizures, but in many cases the patient may retain some form of conscious experience [12,22]. In order to tackle the question of whether consciousness might be retained in absence seizures we need to examine both the notion of consciousness and the question of what criteria (or 'markers') for the ascription of consciousness we should adopt.

There is no illuminating, non-circular definition of consciousness. The best that we can do by way of characterizing the phenomenon is to say that there is 'something it is like' for a creature to be in a conscious state [40]. Conscious states are distinguished from each other on the basis of what it is like to be in them: there is something that it is like to see an object as moving from right to left in front of one, there is something that it is like to taste burnt coffee, and there is something that it is like to recognize an object as a screwdriver.

This 'what it's like' characterization may be adequate from the first-person point of view, but it is clearly highly unsatisfactory from the third-person or scientific perspective. In order to determine whether or not absence seizure patients are conscious – and if so, what particular conscious states they might be in – we need to employ behavioural, cognitive, or neural markers of consciousness.

Here we confront a version of the problem of other minds: which markers should we use in determining whether a creature is conscious [11,26,42]? At present we possess a motley assortment of hunches and intuitions about the kinds of markers that we ought to base our ascription of consciousness on.Some of these markers are likely to be reliable, but others may turn out to be in need of radical revision. This is not an unusual state of affairs, for the possession of robust criteria for the ascription of a property typically follows rather than precedes the development of theoretical models of it. (Consider, for example, our techniques for measuring the presence of a disease.) However, it does mean that there is little that can be said here which is not highly controversial.

Some theorists hold that introspective reportability is a necessary condition on the ascription of consciousness [18,39,44]. If this constraint were to be accepted then few – if any – absence seizure patients would qualify as conscious. However, there is little reason to regard introspective reportability as a *fully general* marker of consciousness. It might be reasonable to require that cognitively intact adult human in the normal waking state will have introspective access to their experience (although even this constraint might be too demanding), but it is surely implausible to adopt introspective reportability as an unrestricted condition on the ascription of consciousness. Such a constraint would prevent us from ascribing consciousness to many non-human animals, infants, and many cognitively impaired adult humans. It may turn out that many non-linguistic creatures are not conscious, but we should not build any such assumption into our methods for studying consciousness.

A more plausible approach to the ascription of consciousness looks to the notion of intentional agency. Consider the contrast between the vegetative state and the minimally conscious state [19,20,30]. In drawing the boundary between these two states physicians lean heavily on appeals to the patient's agentive capacities. Although vegetative state patients open their eyes when awake and make roving eye movements (unlike coma patients), their failure to engage in intentional agency leads most clinicians to regard them as unconscious. Signs of volition indicate that the patient has left the vegetative state and entered the minimally conscious state. Indeed, evidence of voluntary mental imageryis naturally taken as an indicator of consciousness even in patients who would otherwise qualify as being in the vegetative state. A recent study of a 23 year-old patient suggested that she was able to produce sustained mental imagery in response to command. The authors conclude that the patient's

> decision to cooperate with the authors by imagining particular tasks when asked to do so represents a clear act of intention, which confirmed beyond any doubt that she was consciously aware of herself and her surroundings ([41, p. 1402] see also [38]).

One might take issue with the phrase 'beyond any doubt', but these results are certainly highly suggestive of the presence of consciousness. In the same way that limb movement in response to command is naturally taken as a manifestation of consciousness in minimally conscious state patients, so too neural activity that is indicative of mental imagery is naturally taken as a manifestation of consciousness in certain 'vegetative state' patients [43].

A volition-based approach to the ascription of consciousness would suggest that many absence seizure patients are conscious, albeit in a highly attenuated manner. Hughlings-Jackson reported an episode in which a doctor undergoing an absence seizure examined a patient and wrote up the diagnosis in his notes – a diagnosis that accorded with the one that he made when he re-examined the patient the following day [28]. A more recent case concerns a patient who experienced seizures while riding his bicycle to work.

> [The patient] stated that after setting out in the morning he would occasionally find himself riding back home. Clearly, in the space of a relatively short time he had turned around and ridden back home, all the while maintaining his ability to operate a bicycle with the concomitant demands of contact with the environment. Yet he was unable to recollect this period of time [17, p. 114].

It should go without saying that none of these behaviours is *conclusive* evidence of consciousness. The sceptic can always argue that these actions might have been performed unconsciously. She or he might argue that the behaviours seen in absence seizures can be assimilated to the 'automatic' behaviours seen in cognitive neuroscience [34,37] and social psychology [5, 6] – behaviours that appear to occur independently of consciousness.

A full evaluation of this criticism goes beyond the scope of the current paper, but let me make three brief points [9]. Firstly, the evidence of agency outside of consciousness provided by cognitive neuroscience and social psychology is rather more nuanced than appearances suggest. On the face of things, work on the two visual systems appears to undercut the use of agency as a marker of consciousness, for this literature often contrasts conscious ventral-stream 'vision-for-perception' with unconscious dorsal-stream 'vision-for-action.' However, the behavioural repertoire of the dorsal stream itself is extremely limited. The dorsal stream is *not* a homunculus – a "mini-me" that both selects and initiates actions under its own stream – but is more properly thought of on the model of therobotic component of a tele-assistance system: conscious representations in the ventral stream select target objects from the visual array and the dorsal stream carries out the computations required for the assigned action [14,37]. The evidence from social psychology is also liable to over-interpretation. Although theorists often describe subjects as acting on intentions that they are not conscious of, in many cases it seems plausible to suppose that subjects are unaware only of the *origins* of their intentions and remain fully aware of their intentions (or goals) themselves.

Secondly, we need to distinguish different senses in which an action might be said to be unconscious. In common parlance, to do something unconsciously is to do it without being aware of doing it – it is for the action to be unaccompanied by any *conscious monitoring* on the part of the agent. But actions that are not consciously monitored might nonetheless be triggered and guided by conscious perceptual states. Even if absence seizure behaviours are not consciously monitored, they may well be guided by conscious perceptual representations of the patient's environment.

Thirdly, it may be reasonable to regard certain kinds of behaviour as *evidence* of consciousness even if behaviour of that kind can occur in the complete absence of consciousness. In order to function as a marker of consciousness the presence of voluntary agency need only raise the probability of the presence of consciousness. Moreover, it is entirely possible that consciousness is a *systems-based* requirement on intentional activity. Even if cognitive neuroscience and social psychology were to show that intentional behaviours need not involve either conscious perception of the target object nor conscious monitoring of the intention and its execution, this would fall short of demonstrating that intentional, goal-directed behaviour can be carried out by unconscious agents. For all we know, it is only conscious agents that are able to act unconsciously (so to speak).

The case for thinking that consciousness is often retained in absence seizures may not be conclusive but it is far stronger than many casual observers of the phenomenon seem to assume. In my view, it is certainly strong enough for us to be justified in looking to see what absence seizures might be able to teach us about the nature of consciousness.

## 3. Cognitive fragmentation

One of the striking features of absence seizures is the fact that the cognitive impairments that patients suffer can be highly selective. By this I don't mean that patients can lose certain kinds of conscious contents and retain others (although this may indeed be the case), but that patients can lose some of the cognitive operations associated with consciousness whilst retaining others. Consider the following description of the behaviour of a nine-year-old boy undergoing a seizure:

> He had a vacant look on his face. I asked him to close his eyes but he did not comply even though the command was repeated forcefully four times. I then said, 'Ricky, can you hear me?' to which he replied promptly by saying 'yes,' but when the command to close his eyes was again repeated, twice, there was still no compliance. After the attack, questioning revealed that he remembered that I had asked him to close his eyes [22, p. S19].

According to a recent review, the capacity to produce verbal responses to questions is more likely to be disrupted than is the capacity to read or count aloud, and both of these capacities are in turn more likely to be disrupted than is the capacity to encode items in short-term memory, which is in turn more likely to be disrupted than is the capacity to perform simple motor tasks on command [12,29,36].

Reflecting on this fact, Gloor has suggested that asking whether or not the patient has retained or lost consciousness may not be the most helpful question to ask; we would do better, he suggests, to simply focus on describing the patient's cognitive and behavioural profile [22]. That seems to be a sound suggestion from the clinical perspective, but I am interested here in what the science of consciousness can learn from the study of absence seizures. Here, it seems to me, consciousness scientists have a rich and extremely important source of data.

Many accounts of consciousness are premised on the assumption that there is a set of cognitive and behavioural capacities that are intimately connected with consciousness, such that the content of any conscious state is able to 'drive' every member of this set of capacities. This assumption is at the heart of the global workspace approach to consciousness, according to which consciousness just is – or is at least highly correlated with – availability for the 'global' control of cognition and action [3,15,16]. The thought underlying global workspace models is that conscious content – and *only* conscious content – occurs within a workspace which is global in the sense that each of the creature's executive consuming systems have access to the contents of those representations which occur within it. On the face of things, the cognitive selectivity seen in absence seizures poses a challenge to such accounts, for patients appear to have representations whose contents are available to some forms of executive control but not others. In other words, in such patient the global workspace seems to be 'partial' and 'fragmented' rather than fully 'global'.

Broadly speaking, there are two ways in which advocates of global workspace models of consciousness might address this challenge. On the one hand, they couldrestrict the scope of their account to creatures that are in normal background states of consciousness. Although this would not deprive such accounts of interest, it would constitute a severe restriction of ambition: by restricting the account to normal background states of consciousness the global availabilitytheoristwould leave unaddressed the functional nature of consciousness itself.On the other hand, the global workspace theorist could argue that there is a restrictive range of cognitive capacities that remain fully associated even in the context of absence seizures: lose any one member of *this* set of capacities and one loses them all. It is an open question whether or not there is such a set of capacities, but if there is such a set then close attention to the data derived from the study of absence seizures may prove to be invaluable in identifying it. Leaving to one side the question of whether absence seizures might provide challenges to global workspace accounts of consciousness, it is clear that the selectivity ofcognitive impairment seen in such statesprovides an important set of constraints for accounts of the functional role of consciousness.

## 4. Background states and conscious contents

A second set of issues on which absence seizures have the potential to teach us much concerns the relationship between background states of consciousness and the contents of consciousness. Although this relation is an absolutely fundamental one, as yet it remains poorly understood, both conceptually and empirically.

The notion of a background state of consciousness is somewhat intuitive, but it is very difficult to provide a precise definition of the phenomenon. As with many aspects of consciousness, the notion is best introduced by means of examples. Well-known examples of background conscious states would include normal wakefulness, drowsiness, the REM dream state, the hypnotic state, and the minimally conscious state. Background states are often referred to as 'levels' of consciousness; I avoid this term on the grounds that it presupposes that background states can be arranged in a hierarchy, a presupposition that is questionable. To a first approximation we can think of background states as regions within a functional space, the parameters of which govern both the kinds of contents that enter the creature's consciousness and the kinds of cognitive operations that those contents can drive once conscious. Creatures in impaired states of background consciousness will not be able to enjoy the kinds of sophisticated conscious contents that they can enjoy when in the state of normal wakefulness (patients undergoing an absence seizure will not be able to entertain complex thoughts), and those perceptual and sensory states that they do retain may be unable to guide reasoning and decision-making in the way that they normally would be able to. If conscious, patients in the grip of an absence seizure are clearly in a highly restricted background state of consciousness. This background state might not be unique to absence seizures – arguably, it has much in common with the state that minimally conscious state patients are in – but it is nonetheless a state that is remarkably different from the normal waking state in ways which make it very interesting from the point of view of the study of the neural correlates of consciousness.

We can distinguish two kinds of neural correlates of consciousness: the neural correlates of background conscious states ('background-NCCs') and the neural correlates of the contents of consciousness ('content-NCCs') [13]. A wealth of data suggests that content-NCCs can be localized to reasonable fine-grained cortical areas, whereas background-NCCs involve general modifications to sub-cortical structures. By contrast, we know much less about the ways in which content-NCCs and background-NCCs are related to each other: how the mechanisms responsible for a creature's background state of consciousness filter the conscious contents and modulate the functional role of those contents? The dominant methodologies within consciousness science are ill-equipped to tackle this question. One class of studies focuses on content-NCCs. Adopting this approach, theorists hold fixed the creature's background conscious state and look for the neural correlates associated with changes to their conscious contents. Another class of studies seeks to identify those neural markers associated with the transition from one background state to another (say, the transition from the vegetative state to the minimally conscious state), and ignores changes in the contents of consciousness [7,27]. Neither of these strategies is able to identify the ways in which background-NCCs and content-NCCs *interact*. This is where the study of absence seizures might be of some assistance. Identifying the various ways in which the onset and offset of absence seizures disrupts the contents of the patient's consciousness might furnish us with clues about the locus of interaction between content-NCCs and state-NCCs.

A further question concerns the relationship between the background state(s) that characterize absence seizures and those that characterize other disorders of consciousness. The background states that occur in absence seizures appears to have much in common with those seen in many other disorders of consciousness, such as the minimally conscious state [19,20], delirium [21,35], and low levels of general anaesthetic [1,2]. An important – and as yet rather neglected – task for consciousness science is that of mapping the behavioural and neurophysiological relations between these various background states. Not only would such a map provide us with an improved understanding of backgrounds states of consciousness, it would also be an important step towards identifying the behavioural and neurophysiological profile of consciousness itself.

## 5. The unity of consciousness

A third feature of consciousness that may be illuminated by the study of absence seizures is the unity – or, as the case may be, the *dis*unity – of consciousness. There are many conceptions of the unity of consciousness, but for present purposes we can understand the unity of consciousness as follows: a creature has a unified consciousness at time $t$ if, and only if, each of the conscious contents that it has at $t$ is contained within (subsumed by) a single state of consciousness. When consciousness is unified, we can capture exactly what it is like to be the creature in question by appealing to this total conscious state [8,10].

It is an open question whether consciousness is always unified in this sense. Unity of this sort is arguably a feature of normal waking consciousness, but it is possible that the unity of consciousness breaks down in the context of certain impairments of consciousness, such as those seen in absence seizures. Of particular interest to us here is the question of whether the unity of consciousness might break down in the context of seizures with a temporal lobe origin. Such seizures often begin with an 'aura' in which the patient might experience 'phenomenal fragments'. For example, the patient might have a brief feeling of fear, undergo a short hallucination of music, or experience visual imagery. Some auras involve relatively simple perceptual fragments; others take the form of complex, structured multi-modal percepts. Although it is sometimes stipulated that auras take place only in 'that portion of the seizure which occurs before consciousness is lost and for which memory is retained afterwards' [4], there is reason to suspect that aura-like phenomena might persist even after the capacity for memory consolidation has been lost. After all, if isolated bursts of electrical activity can trigger experiential fragments when the patient is able to both report and remember their experiences, perhaps they can also trigger such states when these capacities have been lost. Indeed, certain authors speculate that the patient's very unresponsiveness during temporal lobe seizures might result from the fact his or her attention has been captured by the hallucinations generated by the seizure [22,24].

Consider a patient undergoing a temporal lobe seizure who has multiple phenomenal fragments at the same time. How might these fragments be related to each other? Might some of these phenomenal fragments fail to be unified with each other occur within a single total conscious states, and thus constitute an exception to the general unity of consciousness? Perhaps. Evidence in favour of this proposal would be provided by discovering that the contents of subject's phenomenal fragment were available to quite different cognitive capacities. For example, the contents of one fragment might be available for intentional agency but not memory consolidation, whereas the contents of another fragment might be available for memory consolidation but not intentional agency. This would not *demonstrate* that the two fragments failed to occur within a single phenomenal state, but it would certainly provide evidence for such a claim, and depending on the nature of the capacities in question it might constitute extremely strong evidence for phenomenal fragmentation [8].

## 6. Conclusion

The science of consciousness has much to learn from the study of epileptic absence seizures. The most fundamental question raised by absence seizures is whether – and under what conditions – patients might retain some form of awareness. I have suggested that an approach to the ascription of consciousness based on intentional, goal-directed behaviour gives us reason to think that many patients retain some form of minimal perceptual or bodily awareness, although nodoubt this awareness will be more rudimentary than that seen in the normal waking state. I then examined ways in which the study of absence seizures has the potential to inform our understanding of the functional role of consciousness; the relationship between background states and the contents of consciousness; and the unity of consciousness. Data for theory-building in these domains is not easily obtained, and we should make every effort to use that which might be gleaned from the study of absence seizures.

## Acknowledgements

This research was supported by the *Australian Research Council* (www.arc.gov.au) Discovery Grant DP0984572. I would like to thank Andrea Cavanna, Axel Cleeremans and Jakob Hohwy for their help in preparing this paper for publication.
